# Changes in Hepatic Phospholipid Metabolism in Rats under UV Irradiation and Topically Treated with Cannabidiol

**DOI:** 10.3390/antiox10081157

**Published:** 2021-07-21

**Authors:** Michał Biernacki, Anna Jastrząb, Elżbieta Skrzydlewska

**Affiliations:** Department of Analytical Chemistry, Medical University of Bialystok, A. Mickiewicza 2D, 15-222 Bialystok, Poland; michal.biernacki@umb.edu.pl (M.B.); anna.jastrzab@umb.edu.pl (A.J.)

**Keywords:** UV radiation, cannabidiol, liver, oxidative stress, lipid peroxidation, antioxidants, endocannabinoids, eicosanoids

## Abstract

The liver is a key metabolic organ that is particularly sensitive to environmental factors, including UV radiation. As UV radiation induces oxidative stress and inflammation, natural compounds are under investigation as one method to counteract these consequences. The aim of this study was to assess the effect of topical application of phytocannabinoid-cannabidiol (CBD) on the skin of nude rats chronically irradiated with UVA/UVB, paying particular attention to its impact on the liver antioxidants and phospholipid metabolism. The results of this study indicate that CBD reaches the rat liver where it is then metabolized into decarbonylated cannabidiol, 7-hydroxy-cannabidiol and cannabidiol-glucuronide. CBD increased the levels of GSH and vitamin A after UVB radiation. Moreover, CBD prevents the increase of 4-hydroxynonenal and 8-iso-prostaglandin-F_2α_ levels in UVA-irradiated rats. As a consequence of reductions in phospholipase A2 and cyclooxygenases activity following UV irradiation, CBD upregulates the level of 2-arachidonoylglycerol and downregulates prostaglandin E2 and leukotriene B4. Finally, CBD enhances decreased level of 15-deoxy-Δ-12,14-prostaglandin J2 after UVB radiation and 15-hydroxyeicosatetraenoic acid after UVA radiation. These data show that CBD applied to the skin prevents ROS- and enzyme-dependent phospholipid metabolism in the liver of UV-irradiated rats, suggesting that it may be used as an internal organ protector.

## 1. Introduction

The liver is one of the key metabolic organs in animals, and is responsible for the detoxification of many endogenous and exogenous compounds [1]. Furthermore, it is also the site of the phospholipid metabolism, which, along with cholesterol, is the main components of biological membranes [2], whose phospholipid bilayers perform important structural functions, separating the cell contents from the surrounding environment, creating subcellular organelles and providing platforms for various cell processes [3]. Previous studies have demonstrated that liver pathologies such as non-alcoholic fatty liver disease (NAFLD) and non-alcoholic hepatitis (NASH), contribute to lipid disorders commonly terms as dyslipidemia [4]. Phospholipids containing polyunsaturated fatty acids (PUFAs) are particularly susceptible to the action of reactive oxygen species (ROS), which promote peroxidation based on the classic chain reaction mechanism, resulting in the generation of low-molecular-weight, reactive α,β-unsaturated aldehydes and/or cyclic derivatives such as isoprostanes or neuroprostanes depending on the type of oxidation of the PUFAs underwent [5,6]. Phospholipid oxidation also takes place under the influence of lipolytic enzymes such as: phospholipases, lipoxygenases and cyclooxygenases, which activities are enhanced during oxidative conditions. Consequently, phospholipids are substrates for the generation of bioactive molecules including endocannabinoids and eicosanoids that are involved in signal transduction [7,8].

Endocannabinoids are generated from membrane phospholipids through a multi-step process with the participation of specific enzymes such as: calcium-dependent N-acylphosphatidylethanolamine-hydrolyzing phospholipase D (NAPE-PLD) and diacylglycerol lipase (DAGL) [8]. This biosynthesis takes place on demand, depending on the metabolic needs of cells [9,10]. On the other hand, the synthesis of all eicosanoids is initiated by hydrolysis of phospholipids with the release of PUFAs as a result of the action of cytosolic phospholipase A2 (cPLA2), a widespread and highly conserved esterase. The precursor of eicosanoid synthesis is arachidonic acid, which is metabolized to eicosanoids in oxidative reactions catalyzed by cyclooxygenases (COX-1 and COX-2), lipoxygenases (LOXs) and cytochrome P450 [11]. Arachidonic acid-derived eicosanoids are mainly pro-inflammatory, but also may act as anti-inflammatory mediators [12]. The eicosanoid profile, both in serum and liver, is used to assess, e.g., the development of NAFLD [13].

Metabolic changes in the liver, including phospholipid metabolism, depend on pathophysiological factors affecting the entire body and/or the liver [2,14]. The influence of exogenous chemical factors, including drugs and food ingredients that modify the metabolism of liver phospholipids, has been analyzed [14], but there are also reports showing the influence of physical factors such as UV radiation on changes in liver cell metabolism [15]. Exposure of rat skin to both single and chronic UVA radiation has been found to lead to oxidative stress. After a single dose of UVA radiation, due to the reduced activity of catalase (CAT), glutathione reductase (GSSG-R), and the level of glutathione (GSH), a decrease in the antioxidant effectiveness was observed in the liver [16]. Chronic exposure of animal skin to UVA radiation also resulted in elevated phospholipid peroxidation with enhanced production of malondialdehyde [15]. Moreover, it was shown that UV irradiation enhances inflammation in internal tissues [17].

Due to the fact that UV radiation, as a component of sunlight, accompanies people every day and UVA/UVB radiation is used in phototherapy or as an element of therapy for skin diseases [18], there is a need to identify compounds that may protect internal organs from exposure to UV radiation. One natural compound with antioxidant and anti-inflammatory properties that has been highly researched recently is cannabidiol (CBD)—non-psychoactive phytocannabinoid [19]. In previous studies we showed that CBD after topical application penetrates into the bloodstream and is present in the blood plasma [20]. As a consequence, it is relatively quickly distributed to all organs with a good blood supply, such as the brain, heart, lungs and liver [21]. Currently, the presence and metabolism of CBD in the liver has been demonstrated after oral or intraperitoneal (i.p.) administration [22,23]. CBD can influence phospholipid metabolism, which results in modulation of the levels of endocannabinoids and eicosanoids that are involved in the modification of oxidative stress and inflammation [19,24,25].

Therefore, the aim of this study was to evaluate the effects of topical CBD applied to the skin of nude rats, which were chronically irradiated with UVA or UVB, specifically to observe antioxidant abilities and changes in the hepatic phospholipid metabolism.

## 2. Materials and Methods

### 2.1. Materials

Reagents were obtained from the following sources: CBD from THC Pharm GmbH (Frankfurt, Germany); 8-iso prostaglandin F_2α_-d4 (8-isoPGF_2α_–d4), anandamide-d8 (AEA-d8), 2-arachidonylglycerol-d8 (2-AG-d8), oleoylethanolamide-d4 (OEA-d4), cannabidol-d9 (CBD-d9), leukotriene B4-d4 (LTB4-d4), 5(S)-hydroxy-5Z,8Z,11Z,13E-eicosatetraenoic-5,6,8,9,11,12,14,15-d8 acid (15-HETE-d8) and prostaglandin D2-d4 (PGD2-d4) from Cayman Chemical Company (Ann Arbor, MI, USA); α-tocopherol, retinol, L-glutathione reduced (GSH), glutathione peroxidase (GSH-Px), glutathione reductase (GSSG-R), catalase (CAT), thioredoxin (Trx), β-nicotinamide adenine dinucleotide phosphate hydrate (NADP), β-nicotinamide adenine dinucleotide phosphate, reduced tetra (cyclohexylammonium) salt (NADPH), hexane, butylated hydroxytoluene (BHT), ethanol, O-(2,3,4,5,6-pentafluoro-benzyl) hydroxylamine hydrochloride (PFBHA-HCl), 4-hydroxynonenal (4-HNE) and benzaldehyde-d6 from Sigma-Aldrich (Saint Louis, MO, USA)

### 2.2. Animal Experiment

The experiment was performed using 8–9-week-old male (260–302 g) nude rats (Hsd:RH-Foxn1rnu; Vivari s.c., Warsaw, Poland). Rats were maintained under standard conditions in a 12 h light/12 h dark cycles, with free access to pellets containing a mixture of nutrients such as minerals, fiber and proteins [26]. The animal care and protocols in this study were reviewed and approved by the Local Ethics Committee for Animal Experiments in Olsztyn, Poland (Resolution No. 37/2019 of 26 April 2019).

The rats were divided into 6 different groups of six rats each and skin of the rat’s back was exposed to the physical and chemical treatments:

Control group: petrolatum applied topically (20 min) every 12 h for 4 weeks;
CBD group: CBD (120 mg/kg of body weight (b.w.); 2.5% *w*/*w* in petrolatum) applied topically (20 min) every 12 h for 4 weeks;
UVA group: UVA radiation (365 nm) every 48 h (dose increasing from 0.5 to 5 J/cm^2^) for 4 weeks;
UVA+CBD group: UVA radiation (365 nm) every 48 h (dose increasing from 0.5 to 5 J/cm^2^) and CBD (120 mg/kg of b.w.; 2.5% *w*/*w* in petrolatum) applied topically (20 min) every 12 h for 4 weeks;
UVB group: UVB radiation (312 nm) every 48 h (dose increasing from 0.02 to 2 J/cm^2^) for 4 weeks;
UVB+CBD group: UVB radiation (312 nm) every 48 h (dose increasing from 0.02 to 2 J/cm^2^) and CBD (120 mg/kg of b.w.; 2.5% *w*/*w* in petrolatum) applied topically (20 min) every 12 h for 4 weeks.

In order to evaluate the influence of UV light irradiation a Cosmedico lamp (Stuttgart, Germany), designed for use in phototherapy of human skin diseases was used as a light source. Plastic combs were used to maintain a constant distance of approximately 2 cm between the lamp and the skin. To assure constant conditions were required to reproducibly deliver the specified dose of radiation and at the same time to protect the skin from overheating/burns.

### 2.3. Tissue Preparation

At the end of the experiment, rats were anesthetized by inhalation of isoflurane. The livers were rapidly removed and prepared in two different ways: part of the fresh tissue samples were frozen in liquid nitrogen and pulverized for determination of CBD and its metabolites, endocannabinoids, eicosanoids, 8-iso-prostaglandin-F_2α_ (8-iso-PGF_2α_) and 4-hydroxynonenal (4-HNE); the rest of liver was homogenized in 0.9% NaCl solution—10% homogenates were centrifuged at 20,000× *g* for 15 min at 4 °C and aliquots of the supernatants were taken for the measurement of other biochemical parameters.

### 2.4. Biochemical Studies

#### 2.4.1. Determination of CBD and Its Metabolites Level

CBD and his metabolites (DCBD, 7-OH-CBD, CBD-glucuronide) level was determined using liquid chromatography-tandem mass spectrometry—LC-MS/MS (LC-MS 8060, Shimadzu, Kyoto, Japan) [27]. Pulverized liver samples (100 mg) were extracted by solid phase extraction (SPE) method. Compounds were separated on an Agilent Poroshell 120 EC-C18 analytical column (3.0 × 150 mm; 2.7 µm particle size). Electrospray ionization (ESI) was used in both positive (CBD, DCBD) and negative (7-OH-CBD, CBD-glucuronide) mode for multiple reaction monitoring (MRM) and quantification of each of the analytes. CBD-d_9_ was used as an internal quantification standard. The transitions of precursor to the generated ion were as follows: m/z 315.1→193.0 for CBD; 287.2→135.1 for DCBD; 329.1→261.2 for 7-OH-CBD; 489.3→113.0 for CBD-glucuronide and m/z 324.1→202.2 for CBD-d_9_. CBD and his metabolites levels are expressed in pmol/mg protein and fmol/mg protein.

#### 2.4.2. Determination of Antioxidants Activity/Level

Glutathione peroxidase (GSH-Px, EC.1.11.1.6) activity was assessed spectrophotometrically according to the method of Paglia and Valentine [28]. Enzyme activity was measured at 340 nm and expressed in units representing oxidation of 1 mmol NADPH in 1 min at 25 °C and pH 7.4. Enzyme-specific activity was expressed in U per milligram of protein.

Glutathione reductase (GSSG-R, EC.1.6.4.2) activity was determined spectrophotometrically according to the method of Mize and Langdon [29]. Enzyme activity was measured at 340 nm and expressed in units representing reduction of 1 µmol NADP^+^ in 1 min at 25 °C and pH 7.4. Enzyme-specific activity was expressed in U per milligram of protein.

Catalase (CAT, EC.1.11.1.9) activity was determined spectrophotometrically according to the method of Aebi [30]. Enzyme activity was estimated at 240 nm and expressed in units representing oxidation of 1 µmol hydrogen peroxide in 1 min at 25 °C and pH 7.0. Enzyme-specific activity was expressed in U per milligram of protein.

The thioredoxin reductase (TrxR, EC.1.8.1.9) activity was determined by spectrophotometric measurement using a commercial assay kit (Sigma-Aldrich, St. Louis, MO, USA) [31]. One unit of enzyme activity was specified as the amount that catalyzing the oxidation of 1 µmol NADPH per min at 25 °C and pH 7.0. Enzyme-specific activity was expressed in U per milligram of protein.

Thioredoxin (Trx) level was determined by ELISA measurement (Enzyme Linked ImmunoSorbent Assay) [32]. Liver homogenate samples were added to cover the bottom of a 96-well plate, and incubated overnight at 4 °C with primary antibody against thioredoxin (Abcam, Cambridge, MA, USA). Goat anti-rabbit antibodies were used as a labelled secondary antibody (Dako, Carpinteria, CA, USA). After washing, chromogen substrate was applied (3,3′,5,5′-tetramethyl-benzidine at 0.1 mg/mL) and absorption was measured at 450 nm. The level of Trx was expressed as µmol per milligram of protein.

The level of vitamin A and E was assessed by high-performance liquid chromatography (HPLC) according to the method of DeLeenher [33]. The vitamin A and E were extracted from homogenates using hexane containing 0.025% butylated hydroxytoluene (BHT), dried and diluted in ethanol. The samples were injected into RP-18 column and detected at 294 nm. The levels of vitamins are expressed as µg/mg tissue.

The level of reduced glutathione (GSH) was assessed by capillary electrophoresis (CE) [34]. The system was equipped with an ultraviolet detector set at 200 nm. The separations were performed on a fused-silica capillary on a 40 cm effective length at a constant voltage of 27 kV. The level of GSH was expressed as µmol/mg tissue.

#### 2.4.3. Determination of Phospholipid Metabolism and Mediators

##### Phospholipid Peroxidation Products

Lipid oxidation products were estimated by assessing the level of as 8-isoPGF_2α_ by LC MS/MS (LCMS 8060, Shimadzu, Kioto, Japan) [35] and 4-HNE by GC MS/MS [36]. 8-isoPGF_2α_ was separated on an Agilent Zorbax Extend-C18 analytical column (2.1 × 50 mm, 1.8 µm particle size). Sample was isolated using solid phase extraction (SPE) method. Electrospray ionization (ESI) in negative mode was used for multiple reaction monitoring (MRM) and quantification of 8-isoPGF_2α_. 8-isoPGF_2α_–d_4_ was used as internal standards for quantification. The precursor to the product ion transition was as follows: m/z 353.2→193.1 for 8-isoPGF_2__α_ and 357.2→197.1 for 8-isoPGF_2__α_-d_4_. 4-HNE was analyzed using a 7890 A GC–7000 quadruple MS/MS (Agilent Technologies, Palo Alto, CA USA) equipped with an HP-5 ms capillary column (0.25 mm internal diameter, 0.25 µm film thickness, 30 m length). 4-HNE was derivatized by the addition of O-(2,3,4,5,6-pentafluoro-benzyl) hydroxylamine hydrochloride (PFBHA-HCl) and detected by the selected ion-monitoring (SIM) mode. Quantitation was achieved using an internal standard (benzaldehyde-D6). The ions used were: m/z 333.0 and 181.0 for 4-HNE-PFB-TMS and m/z 307.0 for IS (benzaldehyde-D_6_) derivatives. Levels of 8-iso PGF_2α_ and 4-HNE were expressed in pg/mg tissue.

##### Enzymes Metabolizing Phospholipids

The activity of enzymes involved in the metabolism of phospholipids was examined spectrophotometrically in accordance with the manufacturer’s instructions: cytosolic phospholipase A2 (cPLA2–EC 3.1.1.4) using cPLA2 Assay Kit (Cayman Chemical Company, Ann Arbor, MI, USA) [37], while cyclooxygenase 1 and 2 (COX1/2–EC.1.14.99.1) using a commercial assay kit (Cayman Chemical Company, Ann Arbor, MI, USA) [38]. PLA2 activity was expressed in µmol/min/mg protein, while cyclooxygenases activity was expressed in U per milligram of protein. One unit is defined as the amount of enzyme that will cause the oxidation of 1.0 nmol of TMPD per minute at 25 °C.

##### Eicosanoids

Liquid chromatography-tandem mass spectrometry—LC-MS/MS (LC-MS 8060, Shimadzu, Kyoto, Japan) method was used to identify and quantify the eicosanoids level in rat liver [39]. Pulverized liver samples (100 mg) were extracted by solid phase extraction (SPE) method. Analytes were separated on an Eclipse Plus C18 analytical column (2.1 × 100 mm, 1.8 µm particle size). Electrospray ionization (ESI) in negative mode was used for multiple reaction monitoring (MRM) and quantification of analytes. 15-HETE-d_8,_ LTB_4_-d_4_, and PGD_2_-d_4_ were used as internal standards for quantification. The precursor to the product ion transition was as follows: m/z 335.2→195.1 for LTB_4_, m/z 351.3→271.2 for PGE_2_, 315.2→271.2 for15-d-PGJ_2_, m/z 319→301.2 for 15-HETE, m/z 339.1→197.1 for LTB_4_-d_4_, m/z 355.0→275.3 for PGD_2_-d_4_ and 327.0→226.2 for 15-HETE-d_8_. Levels of eicosanoids were expressed in pmol/g tissue.

##### Endocannabinoids

Liquid chromatography-tandem mass spectrometry—LC-MS/MS (LC-MS 8060, Shimadzu, Kyoto, Japan) method was used to identify and quantify the endocannabinoids level in rat liver [27]. Pulverized liver samples (100 mg) were extracted using solid phase extraction (SPE) method. Analytes were separated on an Agilent Poroshell 120 EC-C18 analytical column (3.0 × 150 mm, 2.7 µm particle size). Electrospray ionization (ESI) in positive mode was used for multiple reaction monitoring (MRM) and quantification of analytes. AEA-d_8,_ 2-AG-d_8,_ and OEA-d_4_ were used as internal standards for quantification. The most sensitive molecular ion→product ion transitions were monitored for quantitation for all endocannabinoids: m/z 348.3→62.15 for AEA, m/z 379.3→287.25 for 2-AG, 326.3→62.0 for OEA, m/z 300.3→62.0 for PEA, m/z 356.2→63.05 for AEA-d8, m/z 387.3→294.0 for 2-AG-d8 and m/z 330.20→66.15 for OEA-d4. Levels of endocannabinoids were expressed in pmol/g tissue for AEA, OEA, PEA and nmol/g tissue for 2-AG.

### 2.5. Statistical Analysis

Data are expressed as mean ± SD, and were analyzed by one-way analysis of variance (ANOVA) followed by post hoc Tukey testing using Statistica software (Statistica 13.3, Stat Soft, Polska, Poland). Values of *p* ≤ 0.05 were considered significant, and only these results were discussed in detail.

## 3. Results

**Figure 1:**
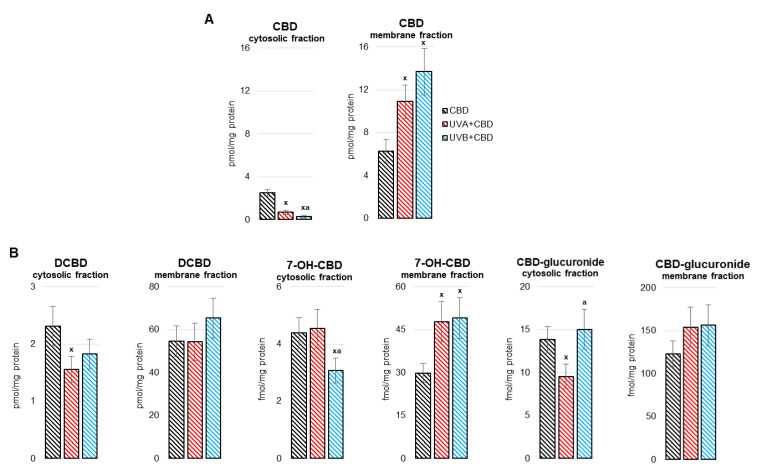
The level of cannabidiol (CBD) (**A**) and CBD metabolites (decarbonylated cannabidiol (DCBD), 7-hydroxycannabidiol (7-OH-CBD), cannabidiol-glucuronide) (**B**) in the liver (cytoplasm and membranes fraction) of nude rats in the following groups: treated with CBD (every 12 h) for 4 weeks; irradiated with UVA (every 48 h) and treated with CBD (every 12 h) for 4 weeks; irradiated with UVB (every 48 h) and treated with CBD (every 12 h) for 4 weeks. The mean values for six rats in each group ± SD are shown: x—differences vs. CBD group, *p* < 0.05; a—differences vs. cells irradiated with UVA (every 48 h) and treated with CBD (every 12 h) for 4 weeks, *p* < 0.05.

The obtained results indicate that cannabidiol (CBD) penetrated through the skin layers into the blood and with it reached the liver, as evidenced by its presence and its metabolites in the liver (Figure 1). Of the CBD metabolites at the quantification level, decarbonylated cannabidiol (DCBD), 7-hydroxycannabidiol (7-OH-CBD) and cannabidiol-glucuronide were found. It was shown that after exposure of rats to UVA/UVB radiation, there was a decrease in the level of CBD in the cytosolic fraction of liver cells, and an increase in the membrane fraction. After exposure to UVA radiation, the levels of DCDB and CBD-glucuronide in the cytosolic fraction decreased. On the other hand, both UVA and UVB radiation increased the level of 7-OH-CBD in the membrane fraction of liver cells, while only UVB radiation led to a decrease of this metabolite in the cytosolic fraction (Figure 1).

**Figure 2:**
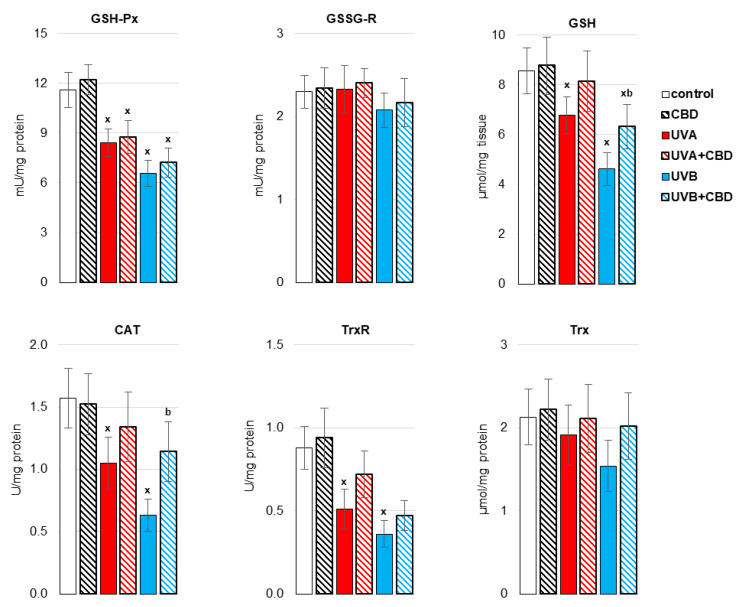
The activity of antioxidant enzymes [glutathione peroxidase (GSH-Px), glutathione reductase GSSG-R), catalase (CAT), thioredoxin reductase (TrxR)] and the level of glutathione (GSH) and thioredoxin (Trx) in the liver of nude rats in the following groups: treated with CBD (every 12 h) for 4 weeks; irradiated with UVA (every 48 h) and treated with CBD (every 12 h) for 4 weeks; irradiated with UVB (every 48 h) and treated with CBD (every 12 h) for 4 weeks. The mean values for six rats in each group ± SD are shown: x—differences vs. control group, *p* < 0.05; b—differences vs. UVB treated group, *p* < 0.05.

**Figure 3:**
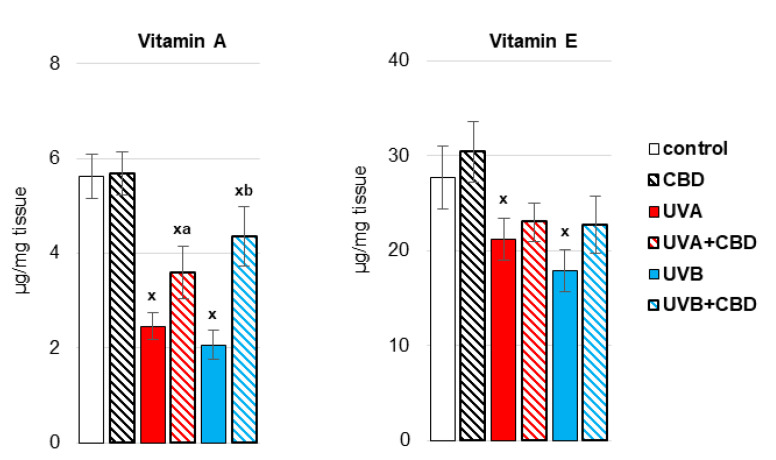
The level of non-enzymatic antioxidants (Vitamin A, Vitamin E) in the liver of nude rats in the following groups: treated with CBD (every 12 h) for 4 weeks; irradiated with UVA (every 48 h) and treated with CBD (every 12 h) for 4 weeks; irradiated with UVB (every 48 h) and treated with CBD (every 12 h) for 4 weeks. The mean values for six rats in each group ± SD are shown: x—differences vs. control group, *p* < 0.05; a—differences vs. UVA treated group, *p* < 0.05; b—differences vs. UVB treated group, *p* < 0.05.

It was previously shown [20] that UVA/UVB irradiation of rats enhances the systemic production of ROS and lowers the antioxidant defense, which is conducive to the formation of oxidative stress. A similar situation is observed at the liver level of rats (Figure 2 and Figure 3). It manifested after exposure of rats to UVA/UVB radiation, lowering the activity of antioxidant enzymes (CAT, TrxR) as well as the activity/level of components of the glutathione-dependent system (GSH-Px; GSH), and the level of other non-enzymatic antioxidants, such as vitamins A and E, cooperating with GSH in protecting biomembranes. Topical administration of CBD partially prevented the impairment of reduced antioxidant protection in the liver of rats exposed to UV radiation. CBD significantly increased the activity of CAT and the levels of some of the analyzed antioxidants, such as GSH and vitamin A, in the liver of rats irradiated with UVB rays, as well as vitamin A in the liver of animals exposed to UVA radiation.

**Figure 4:**
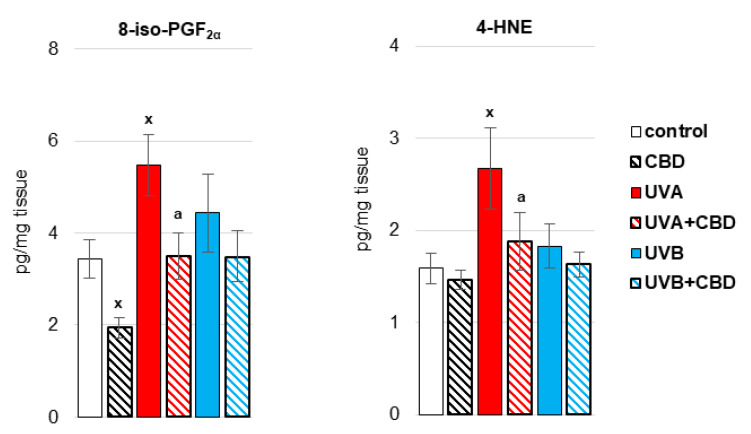
The level of 8-isoprostanes (8-iso-PGF_2α_) and 4-hydroxynonenal (4-HNE) in the liver of nude rats in the following groups: treated with CBD (every 12 h) for 4 weeks; irradiated with UVA (every 48 h) and treated with CBD (every 12 h) for 4 weeks; irradiated with UVB (every 48 h) and treated with CBD (every 12 h) for 4 weeks. The mean values for six rats in each group ± SD are shown: x—differences vs. control group, *p* < 0.05; a—differences vs. UVA treated group, *p* < 0.05.

Antioxidants prevent, reduce or retard the oxidation of compounds, especially high molecular weight compounds such as lipids. Therefore, in the liver of rats irradiated with more penetrating UVA radiation, the effects of oxidative stress were observed in the form of an increase in the level of lipid peroxidation products, including oxidative fragmentation of phospholipids—4-HNE, and the product of oxidative cyclization of phospholipids —8-iso-PGF_2α_ (Figure 4). In contrast, topical application of CBD prevented increase in the level of 4-HNE and 8-iso-PGF_2α_ in the liver of UVA-irradiated rats and decreased the level of 8-iso-PGF_2α_ in control rats.

**Figure 5:**
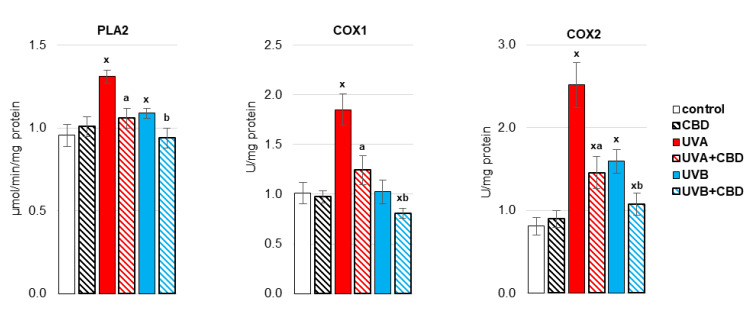
The activity of enzymes involved in the metabolism of eicosanoids (phospholipases A2 (PLA2), cyclooxygenase 1 (COX1), cyclooxygenase 2 (COX2)) in the liver of nude rats in the following groups: treated with CBD (every 12 h) for 4 weeks; irradiated with UVA (every 48 h) and treated with CBD (every 12 h) for 4 weeks; irradiated with UVB (every 48 h) and treated with CBD (every 12 h) for 4 weeks. The mean values for six rats in each group ± SD are shown: x—differences vs. control group, *p* < 0.05; a—differences vs. UVA treated group, *p* < 0.05; b—differences vs. UVB treated group, *p* < 0.05.

**Figure 6:**
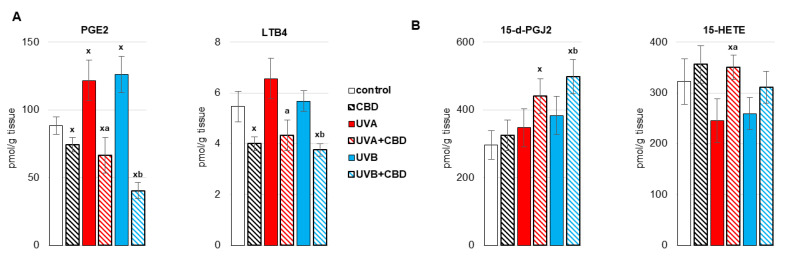
The level of pro-inflammatory eicosanoids (prostaglandin E2 (PGE2), leukotriene B4 (LTB4)) (**A**) and anti-inflammatory eicosanoids (15-deoxy-delta12,14-prostaglandin J2 (15d-PGJ2), 15-hydroxyeicosatetraenoic acid (15-HETE)) (**B**) in the liver of nude rats in the following groups: treated with CBD (every 12 h) for 4 weeks; irradiated with UVA (every 48 h) and treated with CBD (every 12 h) for 4 weeks; irradiated with UVB (every 48 h) and treated with CBD (every 12 h) for 4 weeks. The mean values for six rats in each group ± SD are shown: x—differences vs. control group, *p* < 0.05; a—differences vs. UVA treated group, *p* < 0.05; b—differences vs. UVB treated group, *p* < 0.05.

**Figure 7:**
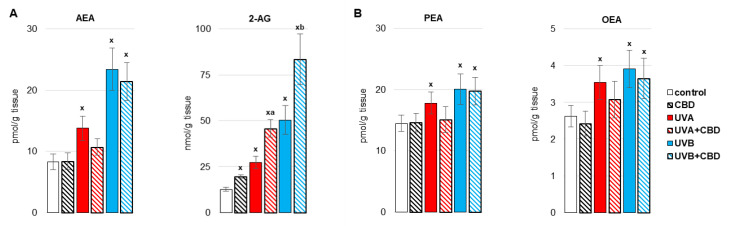
Endocannabinoids level (anandamide (AEA), 2-arachidonylglycerol (2-AG)) (**A**) and related compounds (palmitoylethanolamide (PEA), oleoylethanolamide (OEA)) (**B**) in the liver of nude rats in the following groups: treated with CBD (every 12 h) for 4 weeks; irradiated with UVA (every 48 h) and treated with CBD (every 12 h) for 4 weeks; irradiated with UVB (every 48 h) and treated with CBD (every 12 h) for 4 weeks. The mean values for six rats in each group ± SD are shown: x—differences vs. control group, *p* < 0.05; a—differences vs. UVA treated group, *p* < 0.05; b—differences vs. UVB treated group, *p* < 0.05.

By causing oxidative stress in the liver cells of irradiated rats, UV radiation promoted disorders of liver phospholipid metabolism by changing the activity of enzymes metabolizing phospholipids, including phospholipase A1 as well as isoenzymes of cyclooxygenase (Figure 5, Figure 6 and Figure 7). Both UVA and UVB radiation increased the activity of phospholipase A2 and the induced cyclooxygenase isoform (COX2). On the other hand, the activity of the constitutive isoform COX (COX1) was increased only as a result of UVA irradiation of rats (Figure 5). The above enzymes participate both in the generation of eicosanoids and endocannabinoids. It has been shown that both UVA and UVB radiation significantly increase the level of the pro-inflammatory eicosanoid, PGE2 (Figure 6). Moreover, the levels of two basic endocannabinoids, AEA and 2-AG, as well as related compounds, PEA and OEA, were significantly increased in the liver after UVA/UVB radiation (Figure 7). Topical application of CBD decreased activity of all above enzymes (PLA2, COX1, COX2) in the liver of rats irradiated with UVA and UVB (Figure 5). The consequence of this was a reduction in the level of pro-inflammatory lipid mediators, such as PGE2 and LTB4, in the liver of both control rats and rats irradiated with UVA and UVB radiation (Figure 6). However, the direction of the changes in anti-inflammatory eicosanoids level was the opposite. CBD enhanced the level of 15-d-PGJ2 in the liver of UVB-irradiated rats and 15-HETE in the liver of UVA-irradiated rats. Furthermore, administration of CBD to the skin of control rats and irradiated with UVA/UVB increased only the level of 2-AG in the liver of rats (Figure 7).

## 4. Discussion

On an almost daily basis, individuals are exposed to UV radiation from sunlight. Moreover, UV radiation is quite commonly used in phototherapy to treat skin diseases, such as psoriasis [18]. Earlier studies on the effects of UV radiation on living organisms have focused on metabolic changes in the skin and skin cells after in vivo or in vitro irradiation [40,41,42]. It is known that UVA/UVB radiation intensifies the production of ROS and reduces the level/activity of antioxidants, both in the skin and internal tissues, including blood and liver, which contributes to the formation of oxidative stress, inflammation and consequent metabolic disturbances [15,16]. Therefore, compounds with antioxidant and anti-inflammatory properties are widely searched for in order to prevent disorders caused by UV radiation, both in the skin and internal organs [15,43]. It is especially important to find the possibility of counteracting metabolic disorders of the liver due to its detoxifying role in relation to the whole body.

### 4.1. Effect of UV Radiation on the Metabolism of Liver Phospholipids

In this study, a significant down-regulation of the antioxidant protection of membrane phospholipids in the liver of rats whose skin was chronically exposed to UVA/UVB radiation was found. This reduction is manifested by a decrease in the activity of enzymatic antioxidants such as CAT, TrxR and GSH-Px and the level of non-enzymatic antioxidants including GSH, vitamins A and E. Consequently, the effectiveness of the glutathione-dependent system (GSH-Px; GSH) responsible for the protection of membrane phospholipids decreases, which results in increased lipid peroxidation, as observed in this study. The level of GSH cooperators is also reduced; vitamins A and E, which, by co-working with glutathione, counteract phospholipid modifications. Additionally, Hasegawa [44] observed a decrease in GSH-Px activity and an increase in the level of lipid peroxidation products, but found no changes in superoxide dismutase activity in the livers of mice exposed to a single dose of UVB radiation. A similar situation is observed in our study. Antioxidants prevent, reduce or retard the oxidation of compounds, especially macromolecular compounds such as proteins, lipids and nucleic acids [45], thus preventing metabolic modifications at the cellular level. Therefore, in the liver of rats irradiated with more penetrating UVA radiation, the effects of oxidative stress were observed in the form of an increase in the level of the phospholipid oxidative fragmentation product, 4-HNE, along with the phospholipid oxidative cyclization product—8-iso-PGF_2α_. The electrophilic nature of 4-HNE contributes to its exceptional chemical reactivity and a tendency to form bonds with compounds having nucleophilic groups, including DNA, lipids and proteins [46]. Therefore, 4-HNE acts as an inhibitor of many antioxidants (e.g., thioredoxin, thioredoxin reductase, glutathione peroxidase, catalase) through the formation of adducts with these proteins [46]. 4-HNE has also been observed to stimulate the cytoprotective transcription factor Nrf2, as well as a cooperator of pro-inflammatory transcription factor NFκB [47]. All these metabolic responses promote the disruption of cell signaling, thereby stimulating metabolic dysfunctions, which in the event of proteasomal degradation/autophagy disturbed by oxidative stress, may eventually lead to the accumulation of modified proteins and, consequently, altered cell functionality and/or apoptosis [48].

UV radiation not only promotes ROS-dependent modifications of phospholipids, but also activates enzymes involved in phospholipids metabolism, such as PLA2, COXs, LOXs, leading to their enzyme-dependent oxidation [49,50]. Endogenous bioactive lipid metabolites such as endocannabinoids and eicosanoids are part of a complex network that modulates many cellular processes with pathophysiological consequences [51]. Endogenous free arachidonic acid is a product of phospholipase A2 (PLA2) activity on membrane phospholipids, and this enzyme activity is increased in the liver after irradiation of rats with UVA/UVB radiation. Arachidonic acid undergoes enzymatic metabolism that produces a wide variety of bioactive eicosanoids [51,52]. On the other hand, both cyclooxygenases and lipoxygenases are capable of converting PUFAs, including arachidonic acid, into lipid hydroperoxides [53]. Therefore, in response to UVA and UVB radiation, the biosynthesis of prostaglandin E2 (PGE2) is intensified in the liver, which, through the activation of EP2 and EP4 receptors, reveals a pro-inflammatory effects [54]. Recent studies have shown that the COX2/PGE2-EP2/EP4 axis may be a good target for drugs against liver fibrosis caused by *Schistosoma japonicum* infection [55]. Moreover, the level of other eicosanoids, pro-inflammatory (LTB4) and anti-inflammatory (15-d-PGJ2, 15-HETE), were not changed significantly after UVA/UVB irradiation.

Regardless of the modified generation of eicosanoids under the influence of UVA/UVB radiation, the increased activity of enzymes involved in the metabolism of fatty acids promotes the upregulation of key endocannabinoid representatives such as anandamide (AEA, N-arachidonoethylethanolamine), and 2-arachidonoylethanolamine (2-AG) and the structurally similar oleic acid ethanolamide (OEA), and palmitic acid ethanolamide (PEA) [8], which are synthesized in response to UVA/UVB radiation. Endocannabinoids and their lipid analogues can modulate oxidative stress and inflammation mainly by activating G-protein coupled receptors, among which CB1 is responsible for the generation of ROS and TNF-α, while CB2 and PPARα inhibit ROS/TNF-α production [25]. Importantly, the interaction of ECS and redox homeostasis is particularly involved in the regulation of metabolic tissues, including the liver [56].

### 4.2. Effect of CBD on the Metabolism of Liver Phospholipids

Considering that UV irradiation of rats’ skin promotes the development of oxidative stress causing changes in the metabolism of liver phospholipids, in order to prevent metabolic effects that may affect the whole organism, the effectiveness of phytocannabinoid with antioxidant and anti-inflammatory properties—cannabidiol in counteracting the observed changes in phospholipid metabolism—was investigated. The results of previous studies indicate that cannabidiol applied to the skin penetrates into the blood [57]. However, it is known that cannabinoids are distributed throughout the body along with the blood, which allows CBD to penetrate the liver [58].The results of this study further showed that UVA/UVB radiation promotes the membrane localization of CBD. However, multiomics analysis has shown that CBD penetrates cell membranes, altering the chemical activity of cholesterol and increasing lipid order [59]. Cannabidiol is biotransformed in the liver, where decarbonylated CBD (DCBD) and 7-hydroxycannabidiol (7-OH-CBD) [60,61], observed in our study, are produced by cytochrome P450 (CYP) isoenzymes. 7-OH-CBD is known to be one of the metabolites of CBD generated by CYP2C19 of liver microsomes [61] and this study showed its level increases in the liver membrane fraction after UVA/UVB irradiation. In addition, studies by other authors have shown that two metabolites of cannabidiol: 7-OH-CBD and 7-COOH-CBD have anti-inflammatory effects in relation to mice organism and, depending on the dose, they inhibit the in vitro generation of NO, ROS and TNF-α [62]. Moreover, the dihydrogenated product of 7-OH-CBD—8,9-dihydro-7-OH-CBD also has anti-inflammatory properties indicated in in vitro studies [63]. Both CBD and its phase I metabolites are glucuronidated as part of a phase II response [22].

The results of this study also show the antioxidant effect of CBD at the liver level. This is manifested by the partial prevention of CAT activity and oxidation of non-enzymatic antioxidants, including GSH and vitamin A, which has a positive effect on the protection of membrane phospholipids in which these antioxidants interact. The effectiveness of this action is due to the fact that CBD exhibits much greater antioxidant activity (30–50%) than endogenous antioxidants, such as α-tocopherol or vitamin C [64]. In addition, GSH protection also improves the function of the GSH-Px-GSH system, which manifests itself in the prevention of ROS-dependent lipid peroxidation [65,66]. Consequently, in our studies, we show that topical application of CBD reduces lipid peroxidation, which is revealed by the decreased levels of 8-iso-PGF_2α_ and 4-HNE in the liver of rats exposed to UVA radiation. This type of response to CBD has also been observed in other pathologies, including the liver of C57B/6J mice assessed on the basis of 4-HNE levels [67]. The protective effect of cannabidiol on phospholipids is also evidenced by its complex pharmacological profile in relation to phospholipids, indicating the modulation of the activity of enzymes both directly and indirectly involved in the metabolism of phospholipids with the target generation of endocannabinoids and eicosanoids, which is confirmed by the results of this study. It has been shown that CBD influences the metabolism of arachidonic acid by decreasing the activity of PLA2 and COX1/2, which is confirmed by the literature data [68]. As a consequence, a reduction in the levels of pro-inflammatory and pro-oxidative eicosanoids such as PGE2 and LTB4 is observed. The decrease in PGE2 level may also be related to the identified in this study, increased GSH level. GSH may affect the activity of PGE synthase which controls oxidoreduction of prostaglandin endoperoxide H2 (PGH2) to PGE2, which was observed in the BALF fluid of patients with cystic fibrosis [69]. On the other hand, the reduction of LTB4 levels may have a beneficial effect on liver metabolism, because this eicosanoid promotes oxidative stress, the production of pro-inflammatory cytokines, leukocyte chemotaxis, adhesion and degranulation and the LTB4-BLT2 receptor participates in the growth and proliferation of neoplastic cells [70,71,72]. On the other hand, despite the reduction in the activity of enzymes responsible for the biosynthesis of eicosanoids, the use of CBD resulted in an increase in the level of anti-inflammatory lipid mediators such as 15-d-PGJ2 and 15-HETE. This may be due to the fact that one of the main precursors of 4-HNE is 15-hydroperoxyeicosatetraenoic acid (15-HPETE), which breaks down into different families of more stable compounds, including 15-HETE [73]. Thus, a reduction in the level of 4-HNE may be accompanied by an increase in the level of 15-HETE, which in turn may contribute to the reduction of LTB4 synthesis [74], indicating a potential anti-inflammatory effect of CBD [75]. The observed upregulation by CBD, 15d-PGJ2, a natural PPARγ agonist, also seems to be of particular importance, as this prostaglandin exhibits anti-inflammatory properties by inhibiting the expression of inflammatory cytokines such as MIP-1*β*, TNF-*α* and NOS2 [76]. Moreover, regulating the level of GSH also exhibits antioxidant properties [77]. At the same time, 15d-PGJ2 also shows antitumor activity [78].

By affecting the metabolism of phospholipids, CBD also regulates the level of endocannabinoids, including mainly increased level of 2-AG. Similar relationships were discovered in blood serum and brain cortex after intravenous administration of hemp extract [79]. It can be suggested that this is the result of stimulation of one of the enzymes involved in the biosynthesis of 2-AG, namely diacylglycerol lipase (DAGL) by GSH [80], which is elevated in the liver after CBD application to the skin of UV-irradiated rats. 2-AG is one of the most important endocannabinoids due to both its broad spectrum of action and its high concentration in the liver [10,81]. It has been shown that 2-AG inhibits oxidative stress and inflammation in the liver tissue after hepatic ischemia reperfusion injury which was associated with increased expression of the CB2 membrane receptor responsible for lowering the levels of ROS and TNF-α [82]. Taking into account all the presented results, it can be concluded that CBD improving antioxidant abilities in rat’s liver prevents lipid peroxidation and modulates the metabolism of phospholipids towards anti-inflammatory and antioxidant effects.

## 5. Conclusions

In this study, we discovered for the first time that CBD when applied to the skin reaches the liver, where it undergoes metabolism dependent on the action of physical factors such as UVA/UVB radiation At the same time, it has been shown that CBD application significantly counteracts the reduction of the antioxidant capacity of the liver caused by UV radiation, and thus reduces the level of lipid peroxidation products. In addition, by decreasing the activity of enzymes that metabolize phospholipids and fatty acids, CBD reduces the level of pro-inflammatory and increases the level of anti-inflammatory eicosanoids. The antioxidant and anti-inflammatory effect are supported by the endocannabinoid—2-arachidonylglycerol, the level of which is increased. Thus, applying CBD to the skin may have promising effects in counteracting the negative effects of UV radiation. Thus, the results obtained may constitute a basis for suggesting topical use of CBD to counteract the effects of oxidative stress and inflammation associated with various disease states, especially since CBD has no psychoactive effects.

## Data Availability

The data presented in this study are contained within the article.
